# Mapping chronic disease prevalence based on medication use and socio-demographic variables: an application of LASSO on administrative data sources in healthcare in the Netherlands

**DOI:** 10.1186/s12889-021-10754-4

**Published:** 2021-06-02

**Authors:** Koen Füssenich, Hendriek C. Boshuizen, Markus M. J. Nielen, Erik Buskens, Talitha L. Feenstra

**Affiliations:** 1 RIVM (National Institute for Public Health and the Environment), Centre for Nutrition, Prevention and Health Services, P.O. Box 1, 3720 BA Bilthoven, The Netherlands; 2 Groningen University, UMCG, Department of Epidemiology, Groningen, The Netherlands; 3 Capaciteitsorgaan (Council for Medical Manpower Planning), Utrecht, The Netherlands; 4 Wageningen University and Research, Biometris, Wageningen, The Netherlands; 5 RIVM (National Institute for Public Health and the Environment), Centre for Health and Society, Bilthoven, The Netherlands; 6 NIVEL (Netherlands Institute for Health Srvices Research), Utrecht, The Netherlands; 7 Groningen University, Faculty of Economics and Business, Groningen, The Netherlands; 8 Groningen University, Faculty of Science and Engineering, Groningen Research Institute of Pharmacy, Groningen, The Netherlands

**Keywords:** Disease prevalence, Small area estimates, Machine learning

## Abstract

**Background:**

Policymakers generally lack sufficiently detailed health information to develop localized health policy plans. Chronic disease prevalence mapping is difficult as accurate direct sources are often lacking. Improvement is possible by adding extra information such as medication use and demographic information to identify disease. The aim of the current study was to obtain small geographic area prevalence estimates for four common chronic diseases by modelling based on medication use and socio-economic variables and next to investigate regional patterns of disease.

**Methods:**

Administrative hospital records and general practitioner registry data were linked to medication use and socio-economic characteristics. The training set (*n* = 707,021) contained GP diagnosis and/or hospital admission diagnosis as the standard for disease prevalence. For the entire Dutch population (*n* = 16,777,888), all information except GP diagnosis and hospital admission was available. LASSO regression models for binary outcomes were used to select variables strongly associated with disease. Dutch municipality (non-)standardized prevalence estimates for stroke, CHD, COPD and diabetes were then based on averages of predicted probabilities for each individual inhabitant.

**Results:**

Adding medication use data as a predictor substantially improved model performance. Estimates at the municipality level performed best for diabetes with a weighted percentage error (WPE) of 6.8%, and worst for COPD (WPE 14.5%)Disease prevalence showed clear regional patterns, also after standardization for age.

**Conclusion:**

Adding medication use as an indicator of disease prevalence next to socio-economic variables substantially improved estimates at the municipality level. The resulting individual disease probabilities could be aggregated into any desired regional level and provide a useful tool to identify regional patterns and inform local policy.

**Supplementary Information:**

The online version contains supplementary material available at 10.1186/s12889-021-10754-4.

## Background

Chronic disease prevalence is an important public health indicator. Large differences in disease prevalence have been observed between populations. These are influenced by demographic background, genetics, lifestyle, environmental factors and healthcare policy. As a result, disease prevalence rates strongly vary between small geographic regions [1–3]. Disease mapping may be used to visualize and analyse these differences, which allows for a more efficient allocation of healthcare resources and targeted local healthcare policies [4]. In the Netherlands, chronic disease prevention has been delegated to municipalities, creating demand for disease maps at the municipal level or even at smaller geographic scale, such as neighbourhoods.

At the national level, disease prevalence data is often available from surveys [5–7], hospitalization data [8], GP registries, or insurance claims data [9]. Due to the high costs of collecting data, and medical confidentiality, sample sizes will often be insufficient to create disease maps at a detailed geographic level [10].

As sample sizes are low, researchers have to add extra information to arrive at good small area disease estimates [7]. Often, spatial dependencies are used, borrowing information from geographically proximate regions [11]. An alternative is to use other disease related data available at a regional scale. A frequently used indicator for disease is medication use [12, 13].

Usually disease presence is predicted from medication use based on a theoretical link between disease and medication More recently, studies have explored medication use as a predictor in different types of models [14–16]. These studies use machine learning techniques on training sets with disease diagnosis and medication use data to select medication groups with the highest predictive power. This can outperform prediction based on a theoretical link, since there might be more complex medication use patterns. Using this empirical link between medication use and diagnosis, it is then possible to predict disease probabilities for whom medication use is already known, but disease diagnosis was previously unknown. While these studies showed that medication use is a powerful indicator of disease, they did not investigate to what extend predictions based on medication use can be applied for regional disease prevalence estimates. The current study therefore investigates the added value of medication use and socio-economic variables compared to models using just age and gender to provide prevalence estimates at a small regional scale for diabetes, chronic obstructive pulmonary disease (COPD), coronary heart disease (CHD) and stroke. The performance of such prediction models is analysed as well as the resulting regional patters in The Netherlands.

## Methods

### Data

All data used was accessed and analysed through the System of Social Statistical Datasets (SSD) of Statistics Netherlands. The SSD provides access to multiple administrative data sources, the ability to link pseudo-anonymised data at the individual level, and serves as a Trusted Third Party (TTP). Analyses took place in a secured environment and results can only be exported after control by SSD for privacy and security issues [17]. Dutch law allows the use of electronic health records for research purposes under strict conditions. According to this legislation, neither obtaining informed consent from patients nor approval by a medical ethics committee is obligatory for this type of observational studies containing no directly identifiable data (Dutch Civil Law, Article 7:458).

The population consisted of all those living in the Netherlands on December 31st 2012. Of the 16,779,412 persons recorded, for 16,777,888 persons (99.9%) data was available on date of birth, gender, marital status, municipality, ethnicity, being 1st or 2nd generation immigrant, percentile group of wealth, source of income, percentile group of household income and household composition.

Individual data on medication use were obtained from Medicijntab [18], ‘containing data on persons to whom medicines were dispensed and reimbursed under the statutory basic medical insurance in the year concerned.’ While all Dutch individuals have basic insurance, medications reimbursed differently or sold over the counter are not included in this dataset. All ATC3 (Anatomical Therapeutic Chemical Classification System level 3) codes prescribed to more than 50 persons annually were included. There was no information available on dosage or the number of prescriptions. The only information available was an indicator identifying whether the medication was prescribed or not during a certain year. It was assumed that individuals with no record of a certain ATC3 code did not use this medication in the year of interest.

Diagnosis data was available from two sources, a primary care database and hospital records. When a person was registered in one of the practices participating in the primary care database, the person was included in what we will refer to as the ‘training set’. All Dutch inhabitants are registered in a primary care practice for insurance purposes. The NIVEL primary care database [19] comprises approximately 10% of the Dutch population, with most practices entering during 2002–2006. Diagnostic ICPC-1 codes were registered by general practitioners [20] for all individuals registered to a GP practice participating in the database. The starting year for registration was the date of entry of either the GP into the registry, or the individual into the GP practice.

Clinical and day admissions to hospitals were available from the National Medical Registry [‘Landelijke Medische Registratie’(LMR)] [21] for the years 2002–2012. For 2012 it was estimated that around 25% of admissions were missed by Statistics Netherlands, while previous years had fewer missing cases [21]. Most hospitals reported in ICD9, while in 2012 several hospitals reported in ICD10.

Four important chronic diseases were selected for this study and are defined as in Table 1. If a person had been diagnosed with one of the codes available in Table 1, in either the hospital data (primary and secondary diagnosis) or the primary care data, we considered the person to have the disease/diagnosis category indicated. For stroke and myocardial infarction, having experienced the event in the period covered by the datasets was considered as a chronic disorder for the current study. When neither the hospital records, nor the GP registry indicated a diagnosis, the individual was considered disease free.

**Table 1:** ICD10, ICD9 and ICPC codes [20] per disease

Disease	ICD10	ICD9	ICPC-1
Coronary Heart Disease	I20 – I25	410–414	K74-K76
Stroke	I60 – I69	430–434, 436–438	K90
Diabetes	E10 – E14	250, 648	T90
COPD	J40 – J44	490–492, 496	R91,R95

About 85% of patients in the GP registry could be uniquely linked in the SSD environment to the full set of socio-demographic variables, resulting in a training set of 707,021 individuals, with full diagnostic information being present, as well as complete information on covariates.

### Data analysis

The general approach for this study consisted of three steps. First, we estimated disease probabilities at the individual level. Then, we aggregated these probabilities at the municipality level. Finally, we divided these aggregated numbers by the municipality population size, to find prevalence at the municipality level as the average of the individual probabilities. All analyses were done separately for all four diseases.

Prediction models included, next to ATC3 medication codes, a range of socio-economic variables. Table 2 lists the variables included and their factor levels if appropriate. Adding all interaction terms with age and age^2^, this amounted to 699 potential predictors. Percentile scores for income and wealth, and their second and third degree polynomials were included. Three model variants were distinguished and estimated separately for each disease: The complete model with all 699 predictors, the medication only model, with 182 predictors reflecting ATC3 codes, and the socio-demographics only model with 146 predictors, excluding medication use information.

In order to reduce the number of predictors, a Least Absolute Shrinkage and Selection operator (LASSO) model, with a logit link was fitted using the R package ‘glmnet’ [22], for each of the four diseases separately as dependent variables. The shrinkage parameter was chosen to minimize the misclassification error based on tenfold cross-validation plus one standard error [22], or such that at least 10 predictors were included, whichever of the two resulted in the most variables. Levels of a categorical predictor were considered as separate variables.

Second, based on the total Dutch population, for each municipality, the disease prevalence *P*_*m*_ was computed as the average of the predicted individual disease probabilities ${\hat{p}}_{i\in {n}_m}$ such that ${P}_m=\frac{1}{n_m}\ \sum \limits_1^{n_m}{\hat{p}}_{i\in {n}_m}$. Where *n*_*m*_ is the number of individuals in the municipality with a predicted disease probability.

To assess the internal validity of the resulting prevalence estimates at the municipality level, 5-fold cross validation was used for the LASSO procedure.

Based on the cross-validation, the weighted percentage error (WPE) was computed at the municipality level, ∑_*m* ∈ *M*_*w*_*m*_((*P*_*m*_ − *O*_*m*_)/*O*_*m*_), where m = 1, … *M* is the set of municipalities; *O*_*m*_ is the observed prevalence (percentage) for municipalities in the training set, directly based on the registry data; *P*_*m*_ is the estimated prevalence using either the complete, the medication only or the socio-demographics only model, and *w*_*m*_ is the weight. Weights were computed as subpopulation size in the training set compared to the size of the training set, such that the sum of the weights is 1. For municipalities with few persons in the training set, *O*_*m*_ is zero for several diseases. Hence, only municipalities with more than 500 persons in the training set were included in calculating the WPE.

Next to the unstandardized results, standardized results for age were calculated by applying weights to each individual, before averaging to the municipality level. This estimate allowed to investigate regional differences that remain after correcting for differences in the age of the population. Weights were computed by comparing the age distribution of the municipality to the total Dutch population. Five-year age categories were applied for ages 20–85, while all persons aged below 20 years of age were combined in a single category and also all persons aged 85 years and over were combined in a single category.

## Results

Table 2 shows the characteristics of the training set compared to the total Dutch population. Differences were very small, with a slightly elderly population, and slightly more pensions as source of income in the training set. The first and third quartiles were similar for age, wealth- and income percentile.

**Table 2:** Descriptive statistics in percentages

Variable	Training set	Dutch Population
Mean Age	40.6	40.3
Mean Wealth Percentile	50.3	50.5
Mean Income Percentile	60.7	59.9
Percentage Females	51.1	50.5
Marital Status		
Unmarried	46.5	47.0
Divorced	7.3	7.1
Widowed	5.4	5.2
Married	40.8	40.7
Ethnic Group		
Moroccan	2.0	2.2
Turkish	2.2	2.4
Surinam	2.1	2.1
Netherlands Antilles and Aruba	0.9	0.9
Native	80.2	78.9
Other western	4.0	4.2
Other non-western	8.5	9.4
Immigrant generation		
Native	80.2	78.9
1st generation	9.3	10.7
2nd generation	10.5	10.4
Type of household		
1 person	15.8	16.5
Married couple with children	39.0	39.2
Married couple without children	20.0	19.8
Non-married couple with children	9.1	8.3
Non-married couple without children	6.2	6.3
1 parent with children	8.1	7.9
Institutional	1.2	1.4
Other	0.5	1.4
Source of Income		
Labor	57.2	57.1
Own company	14.8	14.7
Wealth	0.4	0.4
Social benefits	8.2	8.1
Pension	18.3	17.8
Study Grants	0.6	0.8
Other	0.1	0.1
No Income	0.4	1.0

**Fig. 1:**
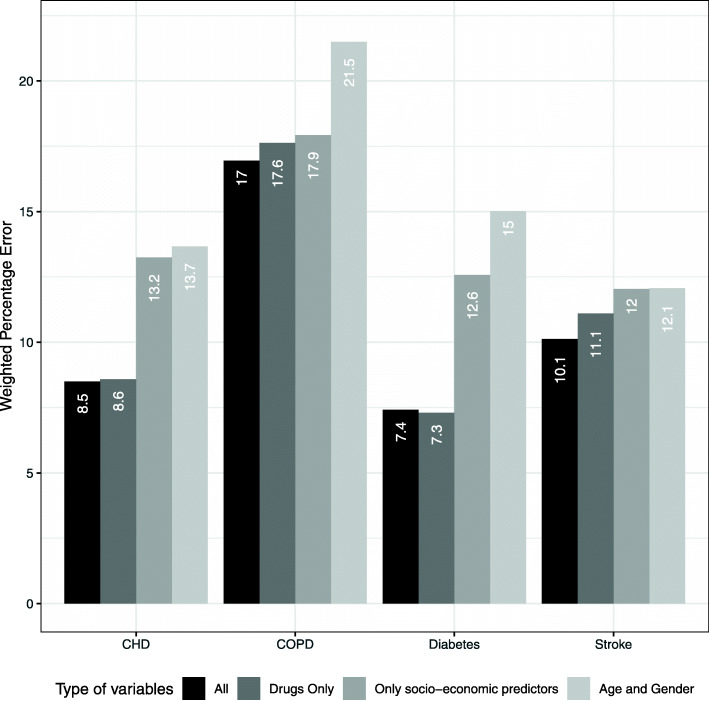
Weighed Percentage Error. Y-axis: Deviation (%) between the estimated prevalence (%) aggregated by municipality and observed prevalence (%) in the training set, weighed by municipality size. X-axis: All: both ATC3 codes and socio-economic predictors, Drugs only: only ATC3 codes, only socio-economic predictors: only socio-economic predictors, or Age and Gender: only age and gender. Created using R version 4.0.2 (https://cran.r-project.org/bin/windows/base/)

Figure 1 shows the AUC for the four diseases and all four models in the training set. An AUC closer to 1 indicates a better fit. A model with only age and gender already fitted well, especially for stroke and CHD. Adding socio-economic variables barely improved the AUC further. Adding medication use, however, improved the AUC for all four diseases. This improvement was largest for diabetes.

**Fig. 2:**
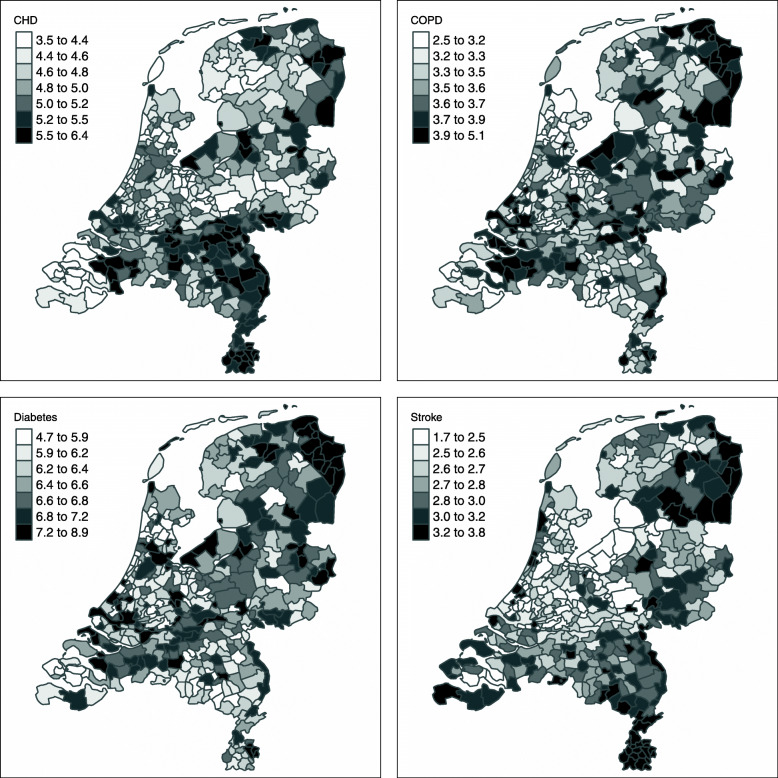
Age-standardized Estimated Municipality Disease Prevalence in the Netherlands**.** Estimated standardized disease prevalence (%) for all Dutch municipalities grouped in septiles. Standardized for age using direct standardization. Created using R version 4.0.2 (https://cran.r-project.org/bin/windows/base/)

Figure 2 shows the fit at the municipality level in the training set. A lower WPE indicates a better fit. As expected, adding more information generally improved the model, and models with only age and gender performed the worst. However, medication use was very predictive for CHD and diabetes, whereas socio-economic variables did not further improve the model. For COPD and stroke, there was a more gradual improvement. Overall, the error for COPD was relatively large, even though adding medication and socio-economic variables decreased the error by several percentage points.

Figure 2 shows the age-standardized maps. Clear regional patterns were observed, which also differed per disease. Appendix 1 shows the unstandardized results, with a slightly different pattern and larger differences. The northern province of Groningen and the south of Limburg showed the highest prevalence both standardized and unstandardized.

## Discussion

In this study we assessed the role of medication use data, demographic information (age and gender) and socio-economic predictors for estimates of disease prevalence probability at the individual level. The resulting predicted probabilities can be used to create maps at any desired level of regional granularity. Maps at the municipality level indeed revealed clear regional patterns that differed by disease. Especially the pattern for stroke stands out and may give important information for capacity building and prevention policy.

Looking at cross-validation results in the training-set, we found that the weighted percentage error at the municipality level from models including both medication use and socio-economic variables was least for diabetes at 6.2%, while it was highest for COPD, with 14.4%.

Adding medication use as predictor improved estimates substantially compared to models that only included socio-economic variables or only included age and gender. This effect was strongest for diabetes, and weakest for stroke. Other researchers estimating disease prevalence rates at small-area level have used mainly age, gender, ethnicity, education or income as predictors, and frequently relied on spatial dependencies to attain estimates for small regions [6, 7, 23, 24]. Our results show that adding medication use improves these estimates.

The current method has several limitations. First, it requires more variables than survey based methods, at least for a training set, while all relevant predictors also have to be available for the entire population for whom estimates are to be obtained. Access to information on medication use, GP and hospital records may be restricted or the data may be difficult to link at the individual level. However, the training set could also be based on alternative sources if these would be more easily available, as long as data on diagnosis as well as medication use and other predictors was available, and the set was representative for the population at large. The main message is that, once a registry is envisioned to be used for prevalence estimates, it is worthwhile considering it as a training set rather than directly extrapolating from the registry diagnoses to the entire population. In this way applying predictors that are also easily available for the entire population to enlarge the precision of regional prevalence data over what can be obtained by simple age and gender based adjustments, appears worthwhile.

Some further limitations in the current study were related to the data sources applied. We had diagnosis data available from GP and hospital sources. However, from the GP records, 85% could be linked individually, and 75% of the hospital records in 2012. To remedy this, we included multiple years of data to capture as much information as possible. Furthermore, we only observed diagnosed cases. Persons with the disease who never went to see a medical professional will not be included in any administrative data source. As such, the prevalence estimates reflect estimates of formally diagnosed disease.

While most of the available data are indicator functions, age, income and wealth are integer and percentile scores respectively. The application of LASSO requires making assumptions with respect to linearity, for which we added polynomials of age, income and wealth. The models only included interactions with age and age^2^, while interactions with socio-economic variables or between ATC groups could be predictive of disease as well.

The current method assumes consistency in prescribing behaviour among medical professionals, and especially GPs in the population of interest. While the Netherlands has centralized prescription guidelines, medical professionals may still treat patients differently. With multiple GPs working in one municipality, this partially averages out. Still, for any estimated differences across municipalities, the question remains whether this is entirely due to differences in underlying health status or partly attributable to differences in prescription pattern across municipalities. Further research separating the two would add to the interpretation of regional differences observed.

Interestingly, applying the method to the Netherlands, we observe clear regional patterns in disease that surpass random noise. We therefore believe our approach can be recommended as a useful tool to monitor and observe regional trends, and identify areas that may require extra attention. For instance, the high prevalence of stroke in the Southern part of the Netherlands may indicate that policy makers should make available sufficient emergency care as well as develop preventive policies in these municipalities.

Regional patterns for the four diseases are also different, indicating that dedicated local policy would be beneficial. Relating such patterns to for instance lifestyle risk factor prevalence and/or socio-demographics could support policy choices in prevention and capacity planning.

## Conclusion

We assessed whether medication use and demographic variables can be used to reliably estimate disease prevalence at the municipal level for stroke, coronary heart disease, diabetes and COPD in the Netherlands. Adding medication use on top of socio-economic variables substantially improved these estimates.

The predicted individual disease probabilities can be aggregated into any desired regional level and provide a useful tool to explore regional patterns and support specific local policies.

## Supplementary Information

**Additional file 1: Appendix Figure 3** Estimated unstandardized disease prevalence (%) for all Dutch municipalities grouped in septiles. Created using R version 4.0.2 (https://cran.r-project.org/bin/windows/base/).

## Data Availability

The data that support the findings of this study are available through the System of Social Statistical Datasets (SSD) of Statistics Netherlands but restrictions apply to the availability of these data, which were used under license for the current study, and so are not publicly available. Data are available through Statistics Netherlands after authorization by the appropriate rightsholders of the data.

## Abbreviations

AUC: Area Under the Receiver Operating Curve
ATC: Anatomical Therapeutic Chemical Classification System
CHD: Coronary Heart Disease
COPD: Chronic Obstructive Pulmonary Disease
GP: General Practitioner
ICD: International Statistical Classification of Diseases and Related Health Problems
LASSO: Least Absolute Shrinkage and Selection Operator
LMR: Landelijke Medische Registratie
NIVEL: Nederlands Instituut voor Onderzoek van de Gezondheidszorg
SSD: System of Social Statistical Datasets
WPE: Weighted Percenatage Error
