# Supplementary figures and images for: Mapping chronic disease prevalence based on medication use and socio-demographic variables: an application of LASSO on administrative data sources in healthcare in the Netherlands
Source: BMC Public Health. 2021 Jun 2;21:1039. doi: 10.1186/s12889-021-10754-4 (PMC8170948; doi:10.1186/s12889-021-10754-4)

CHD

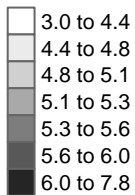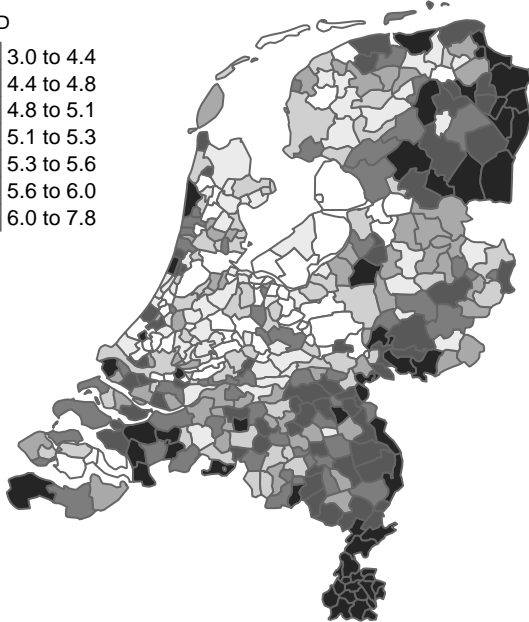

COPD

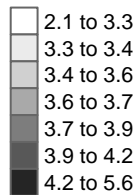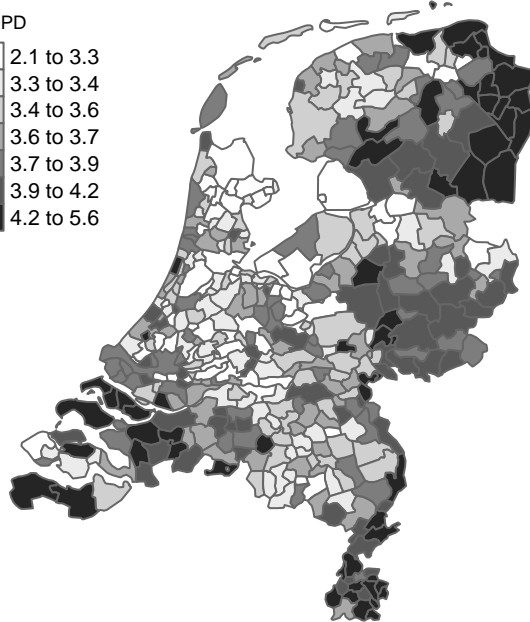

Diabetes

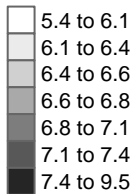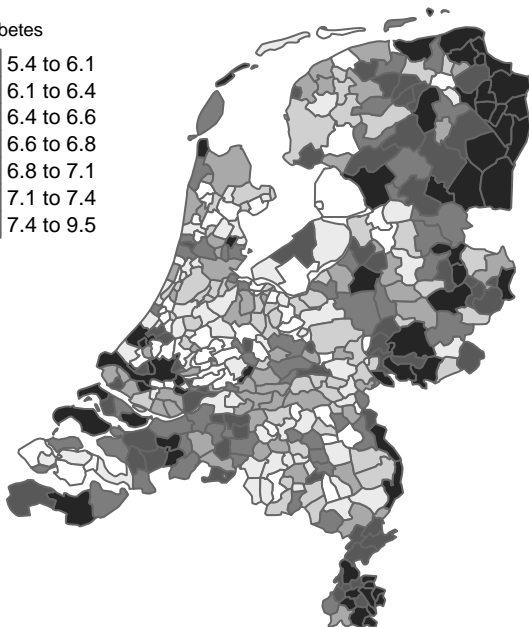

Stroke

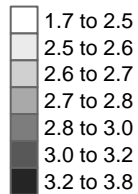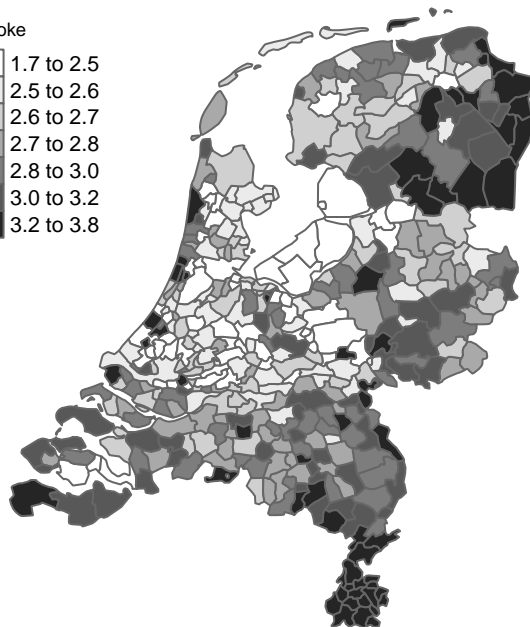

Supplement: Supplementary file 1 — Additional file 1: Appendix Figure 3 Estimated unstandardized disease prevalence (%) for all Dutch municipalities grouped in septiles. Created using R version 4.0.2 (https://cran.r-project.org/bin/windows/base/). [file 12889_2021_10754_MOESM1_ESM.pdf]
